# Controlled Formation of α- and β-Bi_2_O_3_ with Tunable Morphologies for Visible-Light-Driven Photocatalysis

**DOI:** 10.3390/molecules30153190

**Published:** 2025-07-30

**Authors:** Thomas Cadenbach, María Isabel Loyola-Plúa, Freddy Quijano Carrasco, Maria J. Benitez, Alexis Debut, Karla Vizuete

**Affiliations:** 1 Instituto de Energía y Materiales, Departamento de Ingeniería Ambiental, Colegio Politécnico de Ciencias e Ingenierias, Universidad San Francisco de Quito, Quito 170901, Ecuador; marissaloyola@outlook.com; 2 Departamento de Ingeniería Química, Colegio Politécnico de Ciencias e Ingenierias, Universidad San Francisco de Quito, Quito 170901, Ecuador; fquijano@usfq.edu.ec; 3 Departamento de Física, Facultad de Ciencias, Escuela Politécnica Nacional, Ladrón de Guevara E11-253, Quito 170517, Ecuador; maria.benitezr@epn.edu.ec; 4 Departamento Ciencias de la Vida y la Agricultura, Centro de Nanociencia y Nanotecnología, Universidad de las Fuerzas Armadas ESPE, Av. Gral. Rumiñahui s/n, Sangolquí 171103, Ecuador; apdebut@espe.edu.ec (A.D.); ksvizuete@espe.edu.ec (K.V.)

**Keywords:** photocatalysis, emerging pollutants, bismuth oxide, hydrothermal, morphology control

## Abstract

Water pollution caused by increasing industrial and human activity remains a serious environmental challenge, especially due to the persistence of organic contaminants in aquatic systems. Photocatalysis offers a promising and eco-friendly solution, but in the case of bismuth oxide (Bi_2_O_3_) there is still a limited understanding of how structural and morphological features influence photocatalytic performance. In this work, a straightforward hydrothermal synthesis method followed by controlled calcination was developed to produce phase-pure α- and β-Bi_2_O_3_ with tunable morphologies. By varying the hydrothermal temperature and reaction time, distinct structures were successfully obtained, including flower-like, broccoli-like, and fused morphologies. XRD analyses showed that the final crystal phase depends solely on the calcination temperature, with β-Bi_2_O_3_ forming at 350 °C and α-Bi_2_O_3_ at 500 °C. SEM and BET analyses confirmed that morphology and surface area are strongly influenced by the hydrothermal conditions, with the flower-like β-Bi_2_O_3_ exhibiting the highest surface area. UV–Vis spectroscopy revealed that β-Bi_2_O_3_ also has a lower bandgap than its α counterpart, making it more responsive to visible light. Photocatalytic tests using Rhodamine B showed that the flower-like β-Bi_2_O_3_ achieved the highest degradation efficiency (81% in 4 h). Kinetic analysis followed pseudo-first-order behavior, and radical scavenging experiments identified hydroxyl radicals, superoxide radicals, and holes as key active species. The catalyst also demonstrated excellent stability and reusability. Additionally, Methyl Orange (MO), a more stable and persistent azo dye, was selected as a second model pollutant. The flower-like β-Bi_2_O_3_ catalyst achieved 73% degradation of MO at pH = 7 and complete removal under acidic conditions (pH = 2) in less than 3 h. These findings underscore the importance of both phase and morphology in designing high-performance Bi_2_O_3_ photocatalysts for environmental remediation.

## 1. Introduction

The increasing contamination of natural water bodies by persistent organic pollutants has raised significant environmental and public health concerns on a global scale [1,2,3]. Among these pollutants, industrial dyes such as Rhodamine B (RhB) are especially problematic due to their toxicity, chemical stability, and resistance to conventional wastewater treatment methods [4,5,6,7]. As a result, the development of efficient, cost-effective, and environmentally friendly remediation technologies has become an important task in research [6,8].

In this context photocatalysis, and in particular, visible-light photocatalysis, has emerged as a promising green technology for treating polluted water sources [9,10,11]. Here, a semiconductor material facilitates the breakdown of pollutants. When exposed to light with energy equal to or greater than its bandgap, electrons in the valence band (VB) are excited to the conduction band (CB), generating electron–hole pairs. These charge carriers drive redox reactions at the catalyst surface. The excited electrons can reduce oxygen to form superoxide radicals (∙O_2_^−^), while the holes can oxidize water or surface hydroxyl groups to produce hydroxyl radicals (∙OH), both of which are highly reactive and responsible for degrading organic contaminants [9,10,11].

Bismuth oxide (Bi_2_O_3_), an n-type semiconductor, has attracted considerable attention as a visible-light-active photocatalyst due to its narrow bandgap, non-toxicity, chemical stability, and strong absorption in the visible spectrum [12,13,14,15,16]. It also benefits from a relatively low cost and environmental friendliness, making it a suitable candidate for large-scale applications. However, Bi_2_O_3_ is a polymorphic material that exists in several crystalline forms, i.e., monoclinic (α-Bi_2_O_3_), tetragonal (β-Bi_2_O_3_), orthorhombic (ε-Bi_2_O_3_), face-centered cubic (δ-Bi_2_O_3_), body-centered cubic (γ-Bi_2_O_3_), and triclinic (ω-Bi_2_O_3_) [17]. Upon these, the α-phase and the tetragonal β-phase are the most investigated. These polymorphs exhibit distinct electronic structures, optical properties, and photocatalytic behaviors. The α-phase is thermodynamically stable at room temperature, while the β-phase is metastable and typically forms at elevated temperatures. Notably, β-Bi_2_O_3_ has been reported to possess a lower bandgap (~2.1–2.3 eV) than α-Bi_2_O_3_ (~2.6–2.8 eV), enabling enhanced absorption of visible light and often superior photocatalytic activity [14]. Due to its relatively shallow conduction band and the rapid recombination of photoinduced charge carriers, the use of α-Bi_2_O_3_ in photocatalytic applications remains constrained, despite being widely investigated. To address these limitations, many researchers have explored strategies to improve its activity, e.g., through doping with various metal and non-metal elements. While such approaches can enhance performance, they often introduce additional complexities, such as multi-step synthesis procedures, stricter reaction conditions, and higher production costs, making them less appealing for practical, large-scale implementation [18,19,20,21].

In contrast, β-Bi_2_O_3_ offers more promising photocatalytic properties, thanks to its narrower bandgap and stronger absorption in the visible light region. These characteristics make it a compelling alternative for photocatalytic applications. However, despite its advantages, the β-phase has not been as extensively explored. This is largely due to the synthetic challenges associated with producing this metastable polymorph in a controlled and reproducible manner.

In general, the photocatalytic efficiency of Bi_2_O_3_ and other bismuth-based materials is significantly influenced by both its crystalline phase and morphology, i.e., catalyst size and shape [22,23,24,25]. Reducing crystal size leads to an increase in surface area, which can improve the number of active sites and charge carrier separation at the surface. However, a smaller particle size does not always equate to better performance. Nanoscale materials often possess a higher density of surface defects and oxygen vacancies, which often lead to a reduction in efficiency. Furthermore, specific particle shapes have been repeatedly linked to notable differences in photocatalytic behavior, highlighting the importance of morphology [24,26]. This indicates that maximizing photocatalytic activity requires a nuanced balance between phase composition, particle size, and structural morphology.

Various synthetic approaches have been developed to obtain Bi_2_O_3_ with controlled phase and morphology, including sol–gel methods, solid-state reactions, precipitation, and hydrothermal synthesis [13,14,26]. Among these, hydrothermal synthesis has proven to be a particularly effective and versatile technique. It allows precise control over crystal growth under relatively mild conditions and can yield well-defined nanostructures without the need for surfactants or complex processing steps. Furthermore, hydrothermal conditions, such as temperature, time, and precursor concentration, can significantly influence the resulting particle size, shape, and crystallinity.

In recent studies, hydrothermal synthesis followed by calcination has been used to produce phase-pure α- and β-Bi_2_O_3_ [27,28,29,30]. However, while phase formation has been investigated to some extent, less attention has been given to systematically understanding the effect of hydrothermal treatment parameters on morphology development and how these parameters impact the photocatalytic performance of Bi_2_O_3_.

In this work, this gap is addressed by developing a targeted hydrothermal synthesis approach to produce β-Bi_2_O_3_ powders with specific crystalline phases (α and β) and well-controlled morphologies. Special attention is given to tailoring the hydrothermal conditions, specifically reaction temperature and duration, to control the resulting microstructures. Overall, this study provides a comprehensive understanding of how hydrothermal synthesis conditions and subsequent calcination influence both the crystal phase and morphology of Bi_2_O_3_ and how these factors collectively impact its photocatalytic behavior. The findings contribute to the rational design of high-performance photocatalysts for environmental applications and highlight β-Bi_2_O_3_ with flower-like structures as a particularly promising candidate for visible-light-driven degradation of organic pollutants in water treatment systems.

## 2. Results

**Figure 1:**
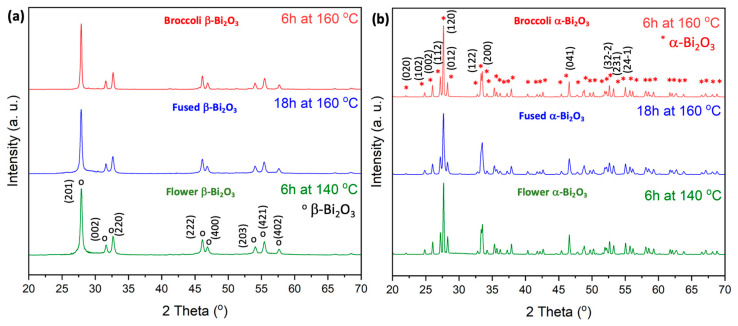
XRD patterns for Bi_2_O_3_ powders. (**a**) Diffractograms of β-Bi_2_O_3_ synthesized at different hydrothermal conditions, calcined at 350 °C. (**b**) Diffractograms of α-Bi_2_O_3_ synthesized at different hydrothermal conditions, calcined at 500 °C.

Building on our previous findings, which showed a clear correlation between calcination temperature and the resulting Bi_2_O_3_ phase, our objective in this work was to deliberately synthesize phase pure α- and β-Bi_2_O_3_ with different morphologies, respectively. To this end, Bi_2_O_3_ powders hydrothermally prepared at 140 °C were subjected to calcination at 350 °C and 500 °C, respectively. The corresponding XRD patterns are shown in Figure 1.

At 350 °C, the material crystallizes into a pure tetragonal β-Bi_2_O_3_ phase, with distinct diffraction peaks observed at 2θ = 28.0, 31.7, 32.8, 46.3, 47.1, 54.3, 55.7, 57.8, 74.6, 75.9, and 77.8. These reflections correspond to the (201), (002), (220), (222), (400), (203), (421), (402), (423), (224), and (442) planes, in agreement with ICDD PDF#27-0050. A complete transformation to the monoclinic α-Bi_2_O_3_ phase is achieved at 500 °C, as confirmed by the dominant diffraction peaks at 2θ = 25.9, 27.0, 27.4, 33.2, 35.1, 37.7, 46.5, 52.6, 58.2, 61.6, and 66.9. These peaks correspond to the (102), (112), (120), (122), (210), (113), (041), (321), (024), (243), and (341) planes of α-Bi_2_O_3_ (ICDD PDF#41-1449).

To investigate the effect of hydrothermal treatment conditions on morphology, additional samples were synthesized via hydrothermal processing at elevated temperatures (140 °C and 160 °C for 6 h) as well as under extended reaction times (140 °C for 18 h). These powders were then calcined under the same set of temperatures, i.e., 350 °C and 500 °C. XRD analyses of these additional samples revealed no deviation in phase behavior: phase pure β-Bi_2_O_3_ formed at 350 °C whereas pure α-Bi_2_O_3_ was obtained at 500 °C, consistent with the results for samples prepared at 140 °C. These findings confirm that the crystalline phase of Bi_2_O_3_ is a result of the calcination temperature, regardless of hydrothermal synthesis parameters such as reaction temperature or duration.

**Figure 2:**
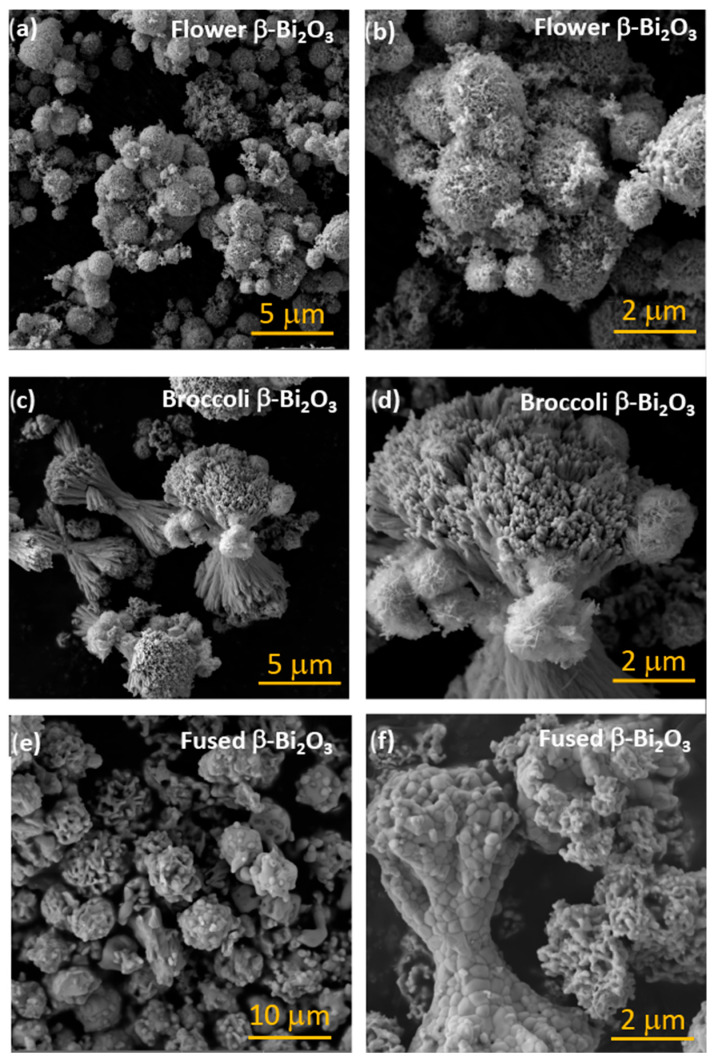
SEM images of β-Bi_2_O_3_. (**a**,**b**) flower-like assemblies of β-Bi_2_O_3_, (**c**,**d**) broccoli-like superstructures of β-Bi_2_O_3_, (**e**) fused, coarsened flower-like assemblies of β-Bi_2_O_3_, (**f**) fused, coarsened broccoli-like superstructures of β-Bi_2_O_3_.

The morphology of the Bi_2_O_3_ samples was investigated using scanning electron microscopy (SEM), which revealed a strong dependence on hydrothermal synthesis parameters, particularly temperature and reaction time. At a hydrothermal treatment temperature of 140 °C for 6 h, the resulting structures consisted of spherical aggregates approximately 1–2 μm in diameter. Higher-magnification SEM images showed that these microspheres were hierarchical flower-like superstructures assembled from thin nanosheets (see Figure 2a,b as well as Figures S1 and S4 in the Supporting Information). These features appeared partially developed, with some structures still in the process of forming fully organized architectures.

When the hydrothermal temperature was increased to 160 °C (for 6h), a pronounced morphological transformation was observed. The flower-like assemblies were largely replaced by elongated rod-shaped structures (see Figure 2c,d). These nanorods, measuring approximately 100–200 nm in diameter and several micrometers in length, aggregated into broccoli-like superstructures, where the tips of the rods formed compact bud-like features reminiscent of a broccoli head. Some residual flower-like formations could still be seen in localized areas, suggesting an intermediate state in the transition process.

For both synthesis temperatures, i.e., 140 °C and 160 °C, increasing the reaction time to 18 h resulted in the loss of well-defined nanostructural features and the emergence of more fused, coarsened morphologies (see Figure 2e,f). This transformation is attributed to a sintering-like process, where prolonged exposure to heat and pressure promotes particle coalescence and structural densification. Specifically, prolonged heating at 140 °C for 18 h led to a structure in which remnants of the flower-like morphology were still visible, but the nanosheet subunits were largely diminished. The resulting structures appeared irregular, with partially fused features and reduced nanoscale definition (see Figure 2e). Due to the high degree of agglomeration and morphological irregularity, this sample was not subjected to further detailed analysis.

Similarly, extending the hydrothermal treatment at 160 °C to 18 h produced a structure broadly resembling that obtained at 160 °C for 6 h. However, the rod-like features became noticeably more agglomerated and fused (see Figure 2f). The broccoli-like superstructures were still identifiable, though the stems and buds were less distinct.

The increased treatment time appeared to promote grain growth and structural coarsening, leading to a partial loss of hierarchical order and finer nanoscale architecture due to prolonged thermal exposure and particle fusion.

It should be noted that the formation of hierarchical flower-like structures is commonly associated with the oriented aggregation and self-assembly of nanosheets under mild hydrothermal conditions. Ethylene glycol and urea, used in the current synthesis, act not only as solvents but also influence nucleation and growth rates, promoting sheet-like subunits that assemble into spherical architectures [31]. When the temperature is increased (e.g., to 160 °C), anisotropic growth is favored, leading to rod-like structures that aggregate into broccoli-like superstructures. Similar transitions have been reported in other Bi-based systems and are often attributed to thermodynamically driven recrystallization processes and Ostwald ripening, which are enhanced at higher temperatures and longer reaction times [26,28,30].

The present work aligns well with reports on temperature-directed morphology evolution in Bi_2_O_3_ and related oxides. For instance, Zhang et al. demonstrated that flower-like Bi_2_O_3_ superstructures could be synthesized under hydrothermal conditions without templates, with morphology strongly depending on precursor chemistry and temperature [31]. Furthermore He et al. reviewed similar temperature-dependent morphological transitions in Bi-based photocatalysts and emphasized the role of reaction kinetics and surface energy minimization in directing particle shape [26].

Importantly, the calcination temperature, i.e., 350 °C vs 500 °C, did not significantly affect the overall morphology in any of the samples (see corresponding morphologies for the α-Bi_2_O_3_ in Figure S1 in the Supporting Information). Regardless of the thermal treatment applied, the key structural features formed during were preserved. These results confirm that the hydrothermal conditions predominantly determine the final morphology of Bi_2_O_3_, while calcination primarily influences phase formation.

To further examine the textural properties of the synthesized Bi_2_O_3_ materials, nitrogen adsorption–desorption isotherm measurements were conducted. These analyses provided insights into specific surface area, pore volume, and average pore size distribution for selected samples. A summary of the results is presented in Table 1, while the corresponding isotherm and pore size distribution plots are included in the Supporting Information (see Figure S2).

All Brunauer–Emmett–Teller (BET) plots show type IV class isotherms with H4 hysteresis loops, which is characteristic of mesoporous materials. The surface area analyses revealed that the highest surface area was observed in the flower-like Bi_2_O_3_ structures, followed by the broccoli-like morphology, and lastly the fused structures. The flower-like and broccoli-like samples also exhibited moderate porosity, as confirmed by Barrett–Joyner–Halenda (BJH) desorption analysis, which indicated a broad pore size distribution ranging from approximately 2 to 60 nm. In contrast, the fused structures appeared to lack significant porosity, consistent with their dense and agglomerated morphology.

Interestingly, the calcination temperature did not significantly affect the textural properties of the materials. Samples containing either β-Bi_2_O_3_ or α-Bi_2_O_3_ phases exhibited comparable surface areas and pore characteristics, indicating that morphological features established during hydrothermal synthesis play a more critical role in determining the final surface area and porosity than the crystalline phase itself.

**Figure 3:**
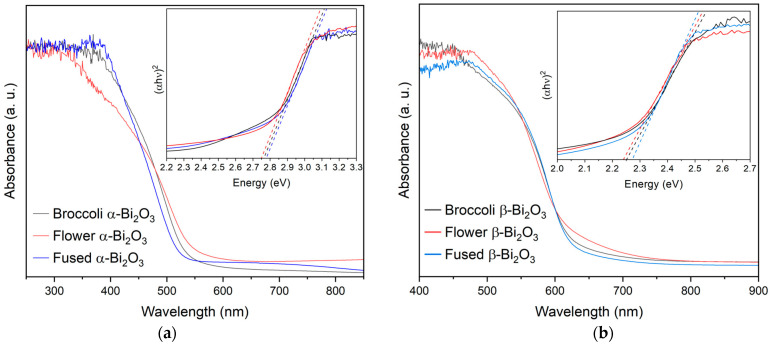
UV–Vis spectra and Kubelka–Munk plots (insets) for α-Bi_2_O_3_ ((**a**) **left**) and β-Bi_2_O_3_ ((**b**) **right**).

The optical characteristics of the synthesized Bi_2_O_3_ materials were examined using diffuse reflectance UV–Vis spectroscopy. Consistent with earlier reports, the UV–Vis diffuse reflectance spectra of the Bi_2_O_3_ samples show clear differences between the two polymorphs [13,32,33,34,35]. The α-Bi_2_O_3_ phase exhibits an absorption edge around 450 nm, while the β-Bi_2_O_3_ phase shows a noticeable red shift, with its absorption edge extending to approximately 550 nm. This shift indicates that β-Bi_2_O_3_ has a stronger capacity to absorb visible light, reinforcing its potential for applications under solar or artificial illumination. To estimate the band gap energies, the reflectance data were converted using the Kubelka–Munk function, and Tauc plots were constructed by plotting the square root of the Kubelka–Munk function against the photon energy. Linear extrapolation of the absorption edge in these plots allowed for the determination of the optical band gaps. Samples composed of pure β-Bi_2_O_3_ exhibited band gap values between 2.24 and 2.27 eV, while those of pure α-Bi_2_O_3_ fell within a higher range of 2.75 to 2.78 eV (see Table 1 and Figure 3). These values align well with those reported in the literature and further support the capability of particularly β-Bi_2_O_3_ for visible and near-UV light absorption, thus making it a promising candidate as a highly responsive material for photocatalytic applications [31,32,36]. The optical differences between the two polymorphs are also visually apparent as β-Bi_2_O_3_ is characterized by an orange-yellow coloration, while α-Bi_2_O_3_ appears white.

**Figure 4:**
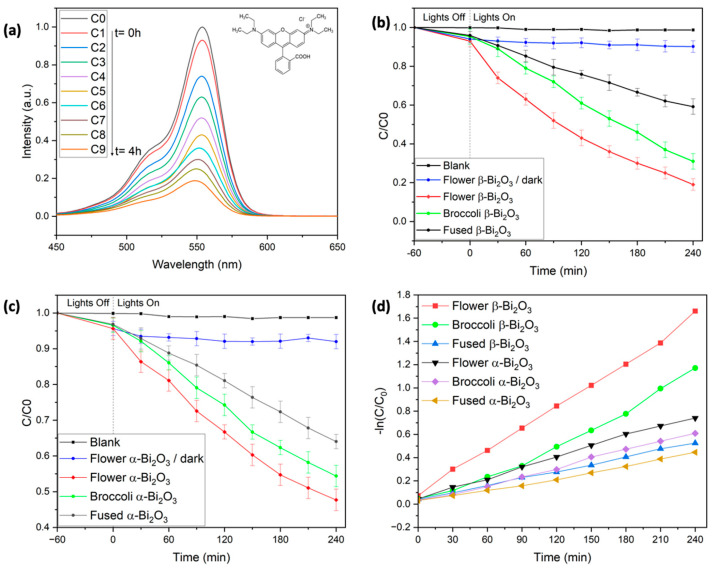
Photocatalytic degradation of Rhodamine B using flower β-Bi_2_O_3_ photocatalysts. (**a**) Evolution of UV–Vis spectra of Rhodamine B during a photocatalytic experiment. (**b**) Concentration of Rhodamine B as a function of reaction time in the presence of various β-Bi_2_O_3_ catalysts. (**c**) Concentration of Rhodamine B as a function of reaction time in the presence of various α-Bi_2_O_3_ catalysts. (**d**) Kinetics of various α- and β-Bi_2_O_3_ catalysts.

To evaluate the photocatalytic activity of the synthesized Bi_2_O_3_ materials, Rhodamine B (RhB), which is a common dye and persistent organic pollutant, was chosen as the model contaminant [37,38,39]. The degradation performance of the phase-pure α- and β-Bi_2_O_3_ samples, each with distinct morphologies, was assessed by tracking the relative concentration (C/C_0_) over time, where C_0_ represents the initial absorbance and C is the absorbance at a given irradiation time (see Figure 4). As expected, RhB showed no measurable degradation when exposed to visible light in the absence of a photocatalyst, even after 4 h of illumination. This was confirmed by the unchanged absorbance peak at 553 nm (Figure 4b), highlighting the need for a photocatalyst to initiate degradation under these conditions.

Prior to light exposure, all reaction mixtures were stirred in the dark for one hour to ensure adsorption–desorption equilibrium. During this period, slight differences in RhB adsorption were observed depending on the Bi_2_O_3_ morphology. The flower-like β-Bi_2_O_3_ samples adsorbed approximately 7% of RhB, about 2% more than the broccoli-like and fused structures. This trend aligns well with the previously measured BET surface areas, where the flower-like structures exhibited the highest surface area [40]. Importantly, control experiments confirmed that RhB concentration remained constant in the absence of light, ruling out significant degradation through dark adsorption or other non-photocatalytic pathways. As in our earlier work with BiFeO_3_-based systems, adsorbed RhB could be effectively removed from the catalyst surface using a water/2-methoxyethanol solvent mixture [41,42].

Under visible-light irradiation (λ = 427 nm and 440 nm), all samples showed a progressive decrease in the characteristic RhB absorption peak at 553 nm, indicating successful photocatalytic degradation. Among the β-Bi_2_O_3_ samples, the flower-like morphology displayed the highest activity, removing approximately 81% of RhB after 240 min. This was followed by the broccoli-like structures, which achieved 69% degradation, and the fused morphology, which reached 41% (see Table 2).

In contrast, α-Bi_2_O_3_ samples showed noticeably lower photocatalytic activity across all morphologies. After 4 h of irradiation, degradation efficiencies were 52% for the flower-like α-Bi_2_O_3_, 45% for the broccoli-shaped structures, and 36% for the fused samples (see Table 2).

These results show that both the crystalline phase and the morphology of Bi_2_O_3_ play key roles in photocatalytic performance. In the degradation of RhB under visible light irradiation, β-Bi_2_O_3_ clearly outperforms α-Bi_2_O_3_. This can be primarily attributed to the lower bandgap of the β-phase (2.24–2.27 eV), allowing for stronger absorption in the visible region, which is in line with previous reports [13,14,32].

Within each phase, a correlation between morphology, surface area, and photocatalytic efficiency is noticeable. The flower-like structures, characterized by hierarchical nanosheet assemblies, consistently exhibit the highest surface areas (∼9.9 m^2^/g), followed by broccoli-like (2.3–3.4 m^2^/g) and fused morphologies (0.5–1.2 m^2^/g). These differences are reflected in the degradation performance. For instance, flower-like β-Bi_2_O_3_ achieved 81% RhB removal after 4 h, whereas broccoli-like β-Bi_2_O_3_ reached 69% and fused structures only removed 41% RhB (see Table 2). This can be explained by the increased surface area, which enhances the number of accessible active sites, facilitates better dye adsorption, and improves charge separation at the catalyst–solution interface. The BET study also shows that flower- and broccoli-like structures are characterized by a porous texture with broad pore size distributions, which further enhance the diffusion and accessibility of reactants to catalytic sites (see Figure S2, Table 1). In contrast, the more compact and fused structures offer fewer active sites, which corresponds to their lower activity. These findings highlight the importance of both crystal phase and morphology in determining the overall photocatalytic performance of Bi_2_O_3_-based materials. The β-phase, especially when synthesized with a flower-like architecture, stands out as the most promising candidate for visible-light-driven degradation of organic pollutants under neutral conditions. Notably, the 81% removal efficiency achieved by the β-Bi_2_O_3_ flower-like sample ranks among the highest reported for RhB degradation under neutral pH and visible-light conditions [22,23,24,25,26,31,32].

To better understand the photocatalytic behavior of the synthesized β-Bi_2_O_3_ materials, the degradation kinetics of Rhodamine B were evaluated using the Langmuir–Hinshelwood (L–H) model. This approach is widely used to describe heterogeneous photocatalytic reactions and accounts for both surface adsorption and reaction dynamics. The rate of degradation can be expressed by Equation (1).

(1) $$r=-\frac{dc}{dt}=\frac{k_{r}K_{c}}{1+K_{c}}$$

where r represents the reaction rate (mg L^−1^ min^−1^), k_r_ is the reaction rate constant (mg L^−1^ min^−1^), K_c_ is the adsorption coefficient (L min^−1^), c is the concentration of RhB (mg L^−1^), and t denotes the irradiation time (min). In systems with relatively low dye concentrations, such as those used in this study, the L–H equation simplifies to a pseudo-first-order kinetic model, as shown in Equation (2). Here, C0 is the initial dye concentration at time t = 0, C is the concentration at a given time t, and k is the pseudo-first-order rate constant (min^−1^).

(2) $$\ln\left(\frac{C}{C_{0}}\right)=-kt$$

According to Equation (2), a plot of ln(C/C_0_) versus irradiation time (t) yields a straight line, confirming that the photocatalytic degradation follows pseudo-first-order kinetics. The slope of the linear fit corresponds directly to the reaction rate constant k, which was calculated for each sample and is summarized in Table 2. These rate constants align well with the overall degradation efficiencies previously observed. Among all tested materials, the β-Bi_2_O_3_ sample with the flower-like morphology exhibited the highest reaction rate, followed by the broccoli-shaped and then the fused structures.

A similar trend in photocatalytic performance was noted within the α-Bi_2_O_3_ series, although the overall rate constants were consistently lower than those of their β-phase counterparts. This difference highlights the superior photocatalytic activity of the β-phase Bi_2_O_3_, which could be attributed to its narrower bandgap and more favorable charge transport properties under visible-light irradiation.

**Figure 5:**
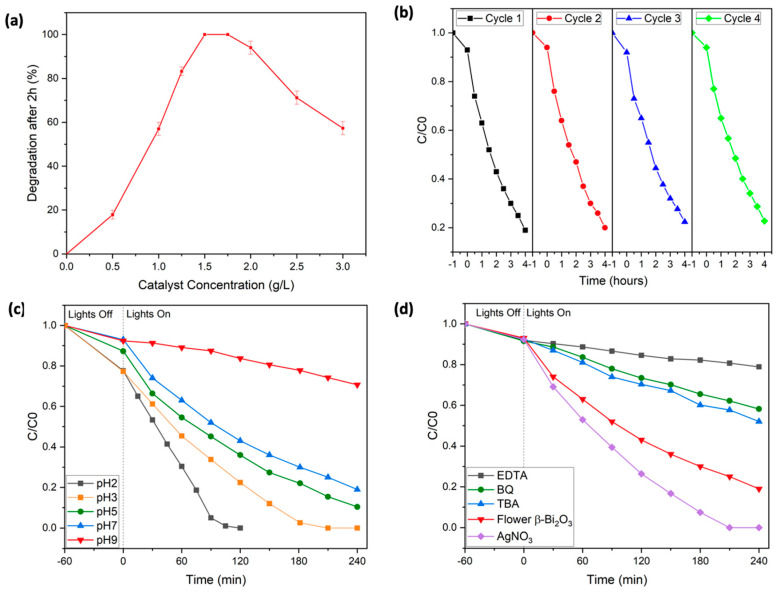
(**a**) Effect of flower β-Bi_2_O_3_ catalyst concentration on the degradation of Rhodamine B. (**b**) Removal of Rhodamine B using flower β-Bi_2_O_3_ in four consecutive cycles. (**c**) Influence of pH on the degradation of RhB by β-Bi_2_O_3_. (**d**) Trapping experiments for the removal of Rhodamine B using flower β-Bi_2_O_3_.

To further optimize the photocatalytic reaction conditions, the flower-like β-Bi_2_O_3_ photocatalyst was selected, as it demonstrated the highest degradation efficiency. First, the influence of various catalyst loadings on the removal of Rhodamine B was investigated. Experiments were conducted using catalyst concentrations ranging from 0 to 3 g/L (see Figure 5a). At lower loadings, degradation was notably slower, likely due to an insufficient number of active sites to support the reaction. As the catalyst concentration increased, performance improved markedly, and complete RhB degradation was achieved within 2 h using 1.5 to 1.75 g/L of the catalyst. However, further increases in catalyst concentration beyond this range led to a drop in efficiency. This decrease is likely due to excessive turbidity in the solution, which hampers light penetration and limits the photocatalytic activity.

The reusability and durability of the flower-shaped β-Bi_2_O_3_ catalyst was evaluated over four consecutive cycles (see Figure 5b). After each cycle, the material was separated via centrifugation; cleaned thoroughly using methoxyethanol, ethanol, and water; and then dried overnight before reuse. Impressively, the catalyst maintained nearly the same activity throughout all four cycles, suggesting excellent long-term stability. X-ray diffraction (XRD) analysis confirmed that the crystalline structure remained unchanged after repeated use, with no evidence of new phases or crystallinity loss (see Figure S3 in the Supporting Information). Notably, the overall morphology, i.e., the flower-like structure of the β-Bi_2_O_3_ photocatalyst, was also still intact after four catalytic cycles, which underlines the overall crystalline and morphological robustness of the synthesized catalyst (see Figure S4 in the Supporting Information).

To further optimize operational conditions, the effect of solution pH on the photocatalytic degradation efficiency was examined by performing experiments across a pH range of 2 to 9 (see Figure 5c). Under alkaline conditions (pH > 7), the degradation efficiency declined noticeably. This can be attributed to electrostatic repulsion between negatively charged hydroxyl groups on the catalyst surface and deprotonated RhB species, which likely reduces dye adsorption. Additionally, at high pH, RhB tends to form dimers, which alters its charge distribution and can interfere with effective degradation. It is also known that, at higher pH levels, hydroxyl radicals—key agents in photocatalysis—react with hydroxide ions to form less reactive species such as superoxide anions (O^−^), further decreasing the oxidative power of the system.

In contrast, lowering the pH improved performance significantly. At acidic conditions, particularly below the second dissociation constant of RhB (pK_s2_ = 3.22), the dye exists mainly in its protonated form, enhancing electrostatic attraction to the catalyst surface. These conditions promoted better adsorption and, consequently, faster degradation [43,44,45]. Notably, at pH = 2, complete removal of RhB was achieved within just 100 min.

To confirm the structural stability of the catalysts under these pH conditions, XRD diffractograms of the samples recovered after photocatalysis at pH = 2 and pH = 9 were recorded. No phase impurities or loss of crystallinity were detected, highlighting the robustness of the Bi_2_O_3_ catalysts throughout the treatment process (see Figure S3).

To better understand how the flower-like β-Bi_2_O_3_ catalyst facilitates the photocatalytic breakdown of RhB and to identify reactive species, a series of radical scavenging experiments were conducted. Tert-butyl alcohol (TBA, 2 mM) was used to inhibit hydroxyl radicals (∙OH), benzoquinone (BQ, 0.5 mM) targeted superoxide radicals (∙O_2_^−^), and ethylenediaminetetraacetic acid (EDTA, 2 mM) acted as a hole (h^+^) scavenger (see Figure 5d). In each case, a notable reduction in photocatalytic efficiency was observed: the presence of TBA limited RhB removal to 48%, BQ further reduced it to 32%, and EDTA had the most pronounced effect, with degradation dropping to just 21%. These outcomes strongly suggest that hydroxyl radicals, superoxide radicals, and photogenerated holes all play crucial roles in the overall reaction mechanism. The effect of introducing silver nitrate (AgNO_3_), a known electron scavenger, was investigated to assess the influence of charge carrier dynamics. The addition of AgNO_3_ significantly enhanced the degradation rate, achieving complete RhB removal within approximately 190 min. This improvement is likely due to silver ions capturing photogenerated electrons, which helps to reduce electron–hole recombination and improve overall charge separation efficiency. Additionally, in the absence of radical scavengers, i.e., in a standard degradation experiment, the characteristic absorption peak of RhB shifted from 553 nm to 548 nm during the degradation process. This blue shift suggests the formation of intermediate products, likely resulting from the stepwise removal of ethyl groups and partial breakdown of the conjugated chromophore system of the dye (see Figure 4a) [46].

**Figure 6:**
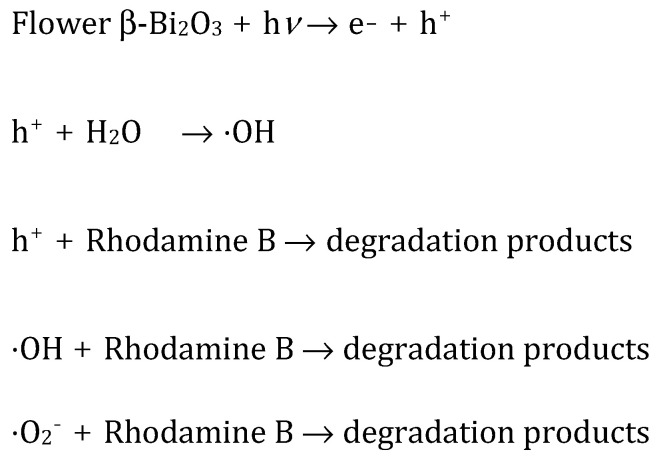
Mechanistic aspects for the photodegradation of RhB using flower β-Bi_2_O_3_.

Altogether, these findings highlight the multifaceted nature of the photocatalytic mechanism, where multiple reactive oxygen species contribute to dye degradation. The beneficial impact of AgNO_3_ further emphasizes the importance of effective charge separation in optimizing photocatalytic performance. These observations align well with previously reported degradation pathways and are summarized in Figure 6 [13,46].

To further evaluate the photocatalytic capabilities of the synthesized flower-like β-Bi_2_O_3_ catalyst, the azo dye Methyl Orange (MO) was selected as an additional model pollutant. Azo dyes are of significant environmental concern due to their toxicity, potential carcinogenicity, and high chemical stability, which render them resistant to conventional wastewater treatment methods [2,3,4,5,6]. As a result, advanced degradation strategies are essential to mitigate their impact before environmental discharge. MO is widely used in textile, pharmaceutical, and research applications and is known for its resistance to photodegradation, particularly in its deprotonated azo form, which predominates at pH values above its pK_a_ (pK_a_ = 3.4). Below this pH, MO adopts a protonated quinone structure that is more reactive and susceptible to degradation. Owing to these characteristics, MO serves as a challenging and representative target for evaluating photocatalytic performance.

**Figure 7:**
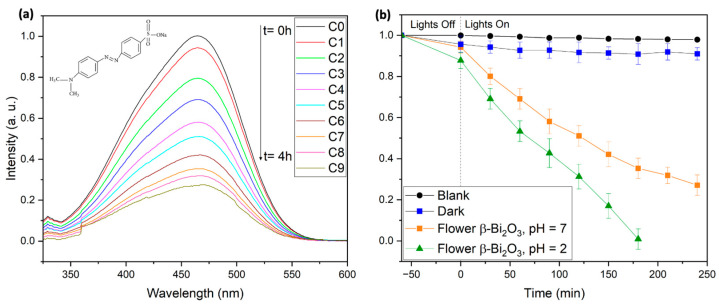
Photocatalytic degradation of Methyl Orange using flower β-Bi_2_O_3_ photocatalysts. (**a**) Evolution of UV–Vis spectra of Methyl Orange during a photocatalytic experiment. (**b**) Concentration of Methyl Orange as a function of reaction time in the presence of various flower β-Bi_2_O_3_.

In this study, the photocatalytic degradation of 10 mg/L MO solutions was carried out under visible-light irradiation (2 × 427 nm and 2 × 440 nm Kessil LED lamps) using flower-like β-Bi_2_O_3_. In the absence of the photocatalyst, MO exhibited only 2.1% degradation after 4 h, confirming its high photostability. In dark conditions, 9% of MO was removed, indicating moderate adsorption by the catalyst. Under visible-light illumination at neutral pH (pH = 7), 73% degradation was achieved within 4 h, highlighting the effectiveness at low catalyst loading and near-neutral conditions (see Figure 7). Furthermore, kinetic analysis of the degradation process showed a pseudo-first-order behavior, consistent with the Langmuir–Hinshelwood model and in agreement with previous reports (see Figure S6) [46]. Notably, at pH = 2, complete degradation occurred in under 3 h. This enhanced activity under acidic conditions can be attributed to the protonated state of MO, which facilitates photocatalytic breakdown (see Figure 7).

These results demonstrate the strong potential of flower-like β-Bi_2_O_3_ as an effective visible-light-active photocatalyst for the removal of stable and toxic azo dyes, offering a promising route for advanced wastewater remediation.

## 3. Materials and Methods

### 3.1. Synthesis of Flower-like Bi_2_O_3_

Bismuth nitrate pentahydrate (Bi(NO_3_)_3_·5H_2_O, 12 mmol, ≥98% purity, Sigma-Aldrich, St. Louis and Burlington, MA, USA, MW = 485.07 g/mol) and citric acid (3 mmol, ≥99.5% purity, Sigma-Aldrich, MW = 192.12 g/mol) were first dissolved in 6 mL of high-purity ethylene glycol (C_2_H_6_O_2_, 99.8%, Sigma-Aldrich). The resulting solution was stirred at 500 rpm for approximately 10 min to ensure homogeneity. Next, 0.54 g of urea (CO(NH_2_)_2_, MW = 60.06 g/mol) and 114 mL of distilled water were added to the reaction mixture. After an additional 15 min of stirring, the prepared solution was transferred into a 300 mL Teflon-lined stainless-steel autoclave.

The sealed autoclave was heated at 140 °C for 6 h to facilitate the hydrothermal reaction. Upon completion, the system was allowed to cool naturally to room temperature. The resulting precipitate was recovered by centrifugation at 1500 rpm for 3 min. To remove residual ions and organics, the solid was washed repeatedly using a 6:1 mixture of distilled water and ethylene glycol. This purification step was performed eight times, with each centrifugation cycle extended by one minute relative to the previous wash.

The purified powder was then dried overnight in a ventilated oven at 80 °C. Finally, thermal treatments were conducted by calcining the dried samples at 350 °C and 500 °C to obtain the β-Bi_2_O_3_ and α-Bi_2_O_3_ products, respectively. Heating was carried out at a controlled rate of 1 °C per minute, while the cooling phase was regulated at a rate of 3 °C per minute.

It is worth noting that the broccoli-like and fused morphologies can be obtained by simply modifying the hydrothermal treatment temperature and duration, as outlined in this study. The subsequent calcination process for these samples remains unchanged.

### 3.2. Characterization Techniques and Equipment

The crystalline structure and phase composition of the synthesized bismuth oxide materials were examined using a Bruker D2 Phaser X-ray diffractometer (Bruker, Billerica, MA, USA) equipped with a Cu Kα radiation source (λ = 1.54184 Å). Diffraction data were analyzed using DIFRACC.EVA software (version 4.3.1.2) to perform a semi-quantitative assessment and detect the presence of any secondary phases. Surface morphology and particle structure were investigated by scanning electron microscopy (SEM) using a TESCAN MIRA 3 field emission microscope (Tescan, Brno, Czech Republic), which was coupled with a Bruker X-Flash 6–30 detector (Bruker, Billerica, MA, USA) offering a resolution of 123 eV for Mn Kα emission.

Diffuse reflectance spectra were acquired with a PerkinElmer UV–Vis spectrophotometer (Perkin Elmer, Waltham, MA, USA) equipped with an integrating sphere, covering a spectral range of 200 to 1000 nm. These reflectance data were subsequently processed using the Kubelka–Munk function to estimate the optical band gap energies of the samples.

To evaluate the textural properties of the materials, nitrogen adsorption–desorption measurements were conducted using a Quantachrome Autosorb IQ 6AG/HOB instrument (Quantachrome, Boynton Beach, FL, USA). The specific surface area of each sample was calculated using the Brunauer–Emmett–Teller (BET) method, while the pore size distribution was determined from the desorption branch of the isotherms via the Density Functional Theory (DFT) method.

### 3.3. Photocatalytic Experiments

The photocatalytic efficiency of the synthesized Bi_2_O_3_ materials was evaluated under ambient conditions using Rhodamine B (RhB) and Methyl Orange (MO) as representative organic pollutants. Aqueous dye solutions were prepared at an initial concentration of 5 mg/L (RhB) and 10 mg/L (MO) at a neutral pH (pH = 7). In a typical experiment, 50 mL of this dye solution was mixed with 50 mg of the Bi_2_O_3_ photocatalyst. To allow for proper adsorption–desorption equilibrium between the dye molecules and the catalyst surface, the mixture was stirred in the dark for one hour prior to light exposure.

Photocatalytic reactions were initiated by irradiating the samples with visible light using four Kessil LED lamps—two emitting at 427 nm (PR160-427) and two at 440 nm (PR160-440)—positioned precisely 10 cm from the center of the reaction vessel to ensure uniform illumination. At regular 30 min intervals, a 5 mL aliquot was withdrawn from the suspension. The photocatalyst was then separated from the solution by centrifugation at 1500 rpm for 3 min.

The residual dye concentration in each sample was quantified by UV–Vis spectroscopy, using the absorbance at maximum wavelength as a reference. Measurements were conducted on a GENESYS 30TM spectrophotometer (GENESYS, Waltham, MA, USA), which utilizes a tungsten-halogen lamp as the light source and a silicon photodiode for detection. Absorbance data were processed and analyzed using Thermo Scientific’s VISIONlite software (Version 5.0), applying the Lambert–Beer law to determine concentration changes throughout the experiment.

## 4. Conclusions

In this study, a facile hydrothermal synthesis was employed to produce phase-pure α- and β-Bi_2_O_3_ powders with well-defined and tunable morphologies. By adjusting hydrothermal treatment parameters, i.e., temperature and duration, precise control over the resulting microstructures was achieved. Synthesis at 140 °C for 6 h yielded hierarchical flower-like superstructures composed of nanosheets, while higher temperatures (160 °C) or prolonged reaction times (18 h) led to the formation of rod-like particles that aggregated into broccoli-like architectures, or, under extreme conditions, into fused and coarsened morphologies. Importantly, phase formation was governed solely by calcination temperature (β-phase at 350 °C, α-phase at 500 °C) and was not influenced by the hydrothermal synthesis conditions.

BET surface area analysis revealed that the flower-like β-Bi_2_O_3_ structures exhibited the highest surface area, correlating with their superior photocatalytic performance. UV–Vis diffuse reflectance spectroscopy confirmed that β-Bi_2_O_3_ consistently exhibited lower bandgap energies (2.24–2.27 eV) than α-Bi_2_O_3_ (2.75–2.78 eV), enabling stronger absorption in the visible-light region. These structural and optical properties translated into enhanced photocatalytic performance. Among all samples, flower-like β-Bi_2_O_3_ showed the highest activity, achieving 81% degradation of Rhodamine B within 4 h under visible light, with complete removal observed under acidic conditions and optimized catalyst concentrations.

To further assess the versatility of the catalyst, Methyl Orange (MO)—a more stable and environmentally persistent azo dye—was selected as an additional model pollutant. Under neutral conditions (pH = 7), the flower-like β-Bi_2_O_3_ catalyst achieved 73% MO degradation within 4 h, and complete degradation was obtained in less than 3 h at pH = 2. These results highlight the capability of the material to degrade structurally diverse organic contaminants. Kinetic studies confirmed pseudo-first-order behavior for both dyes, consistent with the Langmuir–Hinshelwood model. Reusability tests further confirmed the excellent stability of the flower-like β-Bi_2_O_3_ over multiple cycles, with no significant changes in phase or morphology. Radical scavenging experiments indicated that hydroxyl radicals, superoxide radicals, and photogenerated holes play key roles in the photocatalytic mechanism.

Overall, this work emphasizes the importance of morphology and phase control in designing efficient Bi_2_O_3_-based photocatalysts. The results provide valuable insights for the development of robust and effective materials for visible-light-driven degradation of persistent organic pollutants in water treatment applications.

## Figures and Tables

**Table 1:** Overview of surface area, pore size, and bandgaps of the synthesized Bi_2_O_3_ powders.

	Flower Shaped		Broccoli Shaped		Fused Aggregates	
	α-Bi_2_O_3_	β-Bi_2_O_3_	α-Bi_2_O_3_	β-Bi_2_O_3_	α-Bi_2_O_3_	β-Bi_2_O_3_
Surface Area (m^2^/g)	9.93	9.95	2.33	3.44	1.24	0.47
Pore Size (nm)	4–50	15–80	10–35	10–30	--	-
Bandgap (eV)	2.75	2.24	2.76	2.25	2.78	2.27

**Table 2:** Overview of the total degradation of Rhodamine B by various Bi_2_O_3_ photocatalysts and rate constants for the degradation reactions.

	α-Bi_2_O_3_			β-Bi_2_O_3_		
Morphology	Flower	Broccoli	Fused	Flower	Broccoli	Fused
Total degradation % after 4 h (at pH = 7)	52	45	36	81	69	41
Rate constant (10^−3^ min^−1^)	3.0	2.5	1.7	6.4	4.7	2.0

## Data Availability

The original contributions presented in this study are included in the article/Supplementary Materials. Further inquiries can be directed to the corresponding author(s).

## Supplementary Materials

The following supporting information can be downloaded at: https://www.mdpi.com/article/10.3390/molecules30153190/s1, Figure S1. SEM images of α-Bi_2_O_3_. (a) flower-like assemblies of α-Bi_2_O_3_, (b) broccoli-like superstructures of α-Bi_2_O_3_. (c) fused, coarsened flower-like assemblies of α-Bi_2_O_3_, (d) fused, coarsened broccoli-like superstructures of α-Bi_2_O_3._ Figure S2: N_2_ adsorption–desorption isotherm and pore size distribution (inset); (a) Flower α-Bi_2_O_3_, (b) Flower β-Bi_2_O_3_, (c) Broccoli β-Bi_2_O_3_, (d) Broccoli α-Bi_2_O_3_, (e) Fused α-Bi_2_O_3_, (f) Fused β-Bi_2_O_3._ Figure S3: XRD patterns for Flower β-Bi_2_O_3_ before photocatalysis and after 5 catalytic cycles. Figure S4: SEM images of Flower β-Bi_2_O_3_ after 4 photocatalytic cycles. Figure S5: Kinetics of photocatalytic degradation of RhB using different catalysts. Figure S6: Kinetics of photocatalytic degradation of MO using flower β-Bi_2_O_3_.

## Abbreviations

The following abbreviations are used in this manuscript:

RhB	Rhodamine B
TBA	Tert-butyl alcohol
BQ	Benzoquinone
EDTA	ethylenediaminetetraacetic acid
BET	Brunauer–Emmett–Teller
MO	Methyl Orange
