# Supplementary material for: Controlled Formation of α- and β-Bi2O3 with Tunable Morphologies for Visible-Light-Driven Photocatalysis
Source: Molecules. 2025 Jul 30;30(15):3190. doi: 10.3390/molecules30153190 (PMC12348981; doi:10.3390/molecules30153190)
Supplement: Supplementary file 1 [file molecules-30-03190-s001.zip › molecules-3735531-supplementary.pdf]

Supporting Information

Controlled Formation of α- and β-Bi_2O_3 with Tunable Morphologies for Visible-Light-Driven Photocatalysis

Thomas Cadenbach[1,*], María Isabel Loyola-Plúa[1], Freddy Quijano Carrasco,[2] Maria J. Benitez[3], Alexis Debut[4] and Karla Vizuete[4]

[1] Universidad San Francisco de Quito, Colegio Politécnico de Ciencias e Ingenierias, Departamento de Ingeniería Ambiental, Instituto de Energía y Materiales, Quito, Ecuador
[2] Universidad San Francisco de Quito, Colegio Politécnico de Ciencias e Ingenierias, Departamento de Ingeniería Química, Quito, Ecuador
[3] Departamento de Física, Facultad de Ciencias, Escuela Politécnica Nacional, Ladrón de Guevara E11-253, Quito 170517, Ecuador
[4] Centro de Nanociencia y Nanotecnología, Universidad de las Fuerzas Armadas ESPE, Av. Gral. Rumiñahui s/n, Sangolquí, PO Box 171-5-231B, Ecuador

* Correspondence: tcadenbach@usfq.edu.ec

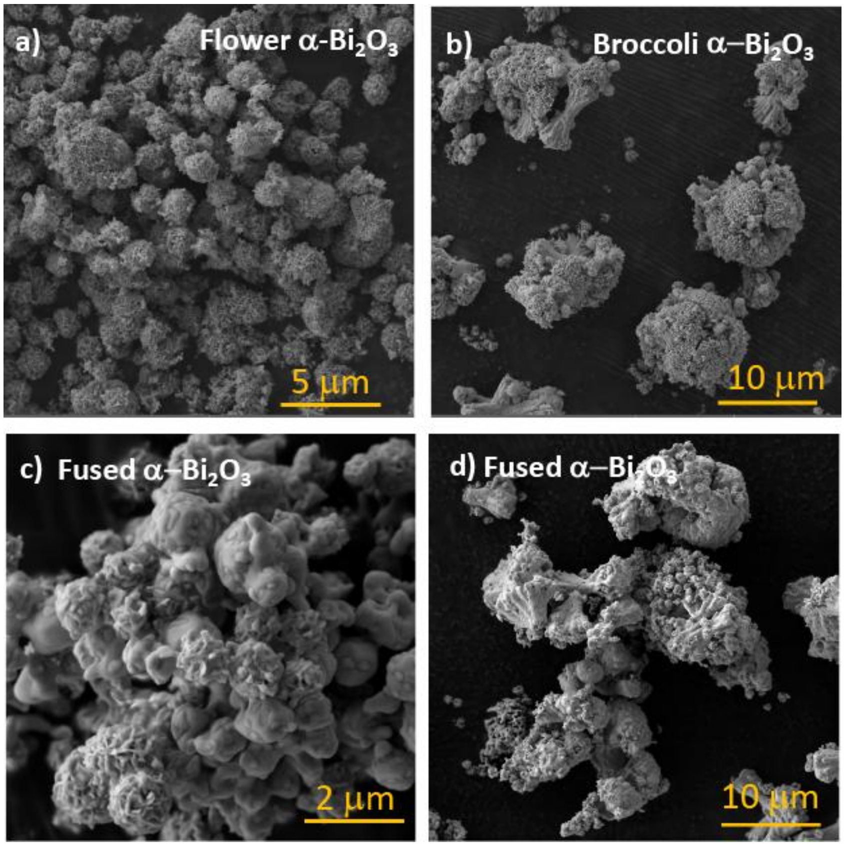

Figure S1. SEM images of α-Bi_2O_3. a) flower-like assemblies of α-Bi_2O_3, b) broccoli-like superstructures of α-Bi_2O_3. c) fused, coarsened flower-like assemblies of α-Bi_2O_3, d) fused, coarsened broccoli-like superstructures of α-Bi_2O_3

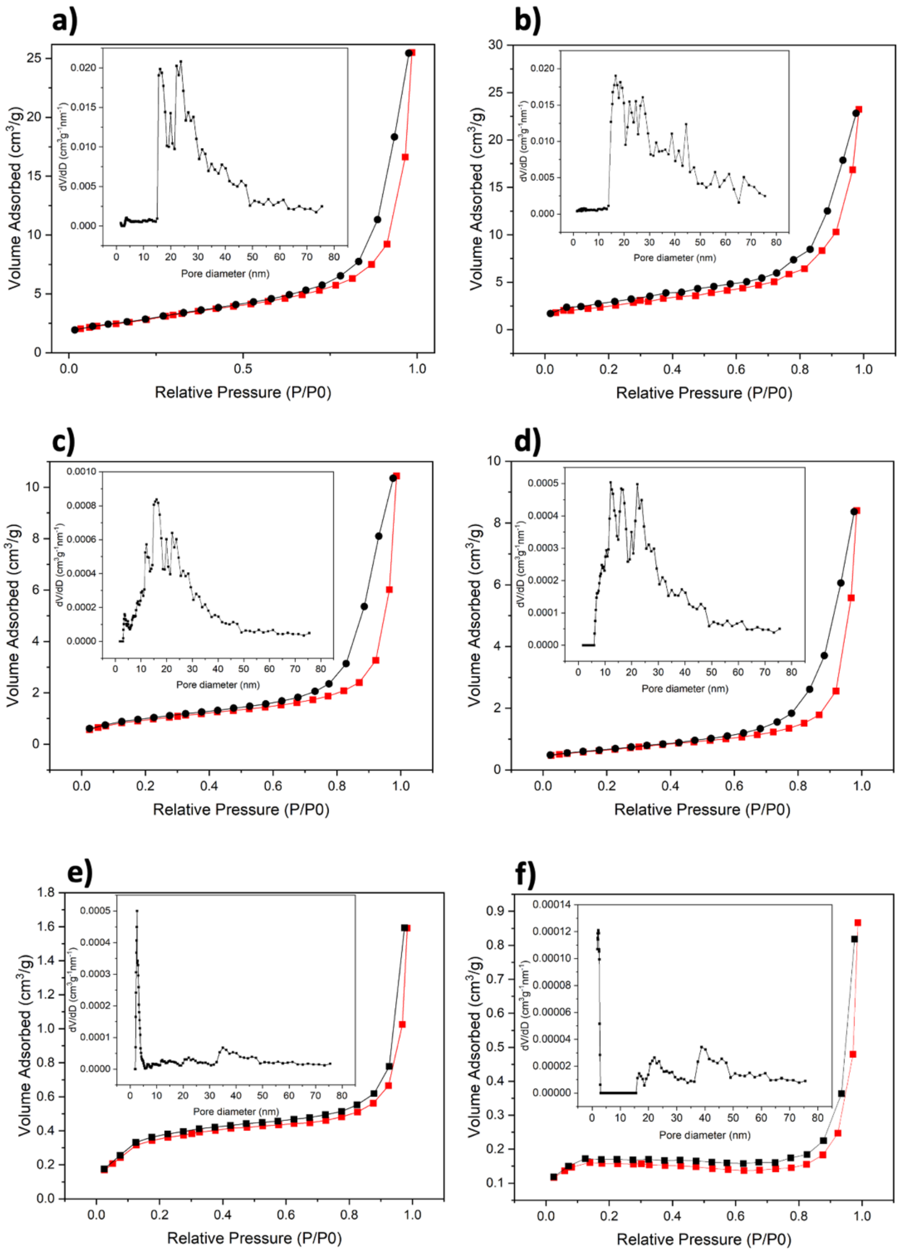

Figure S2: N_2 adsorption–desorption isotherm and pore size distribution (inset); a) Flower α-Bi_2O_3, b) Flower β-Bi_2O_3, c) Broccoli β-Bi_2O_3, d) Broccoli α-Bi_2O_3, e) Fused α-Bi_2O_3, f) Fused β-Bi_2O_3

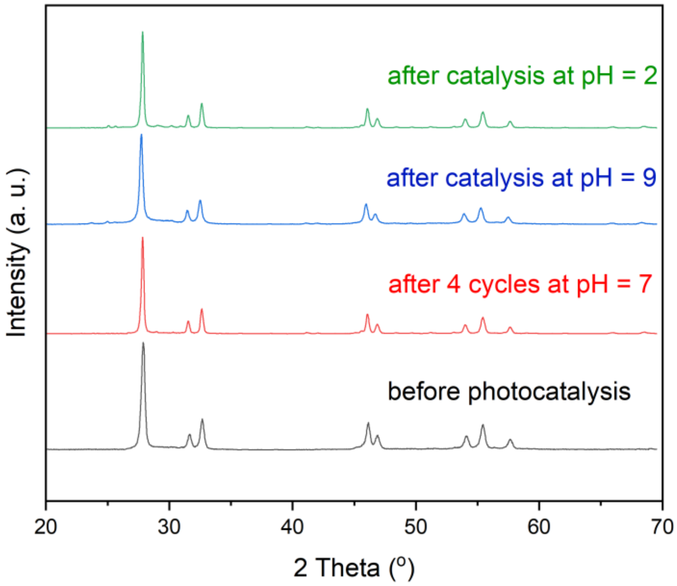

Figure S3: XRD patterns for Flower β-Bi_2O_3 before photocatalysis and after 5 catalytic cycles.

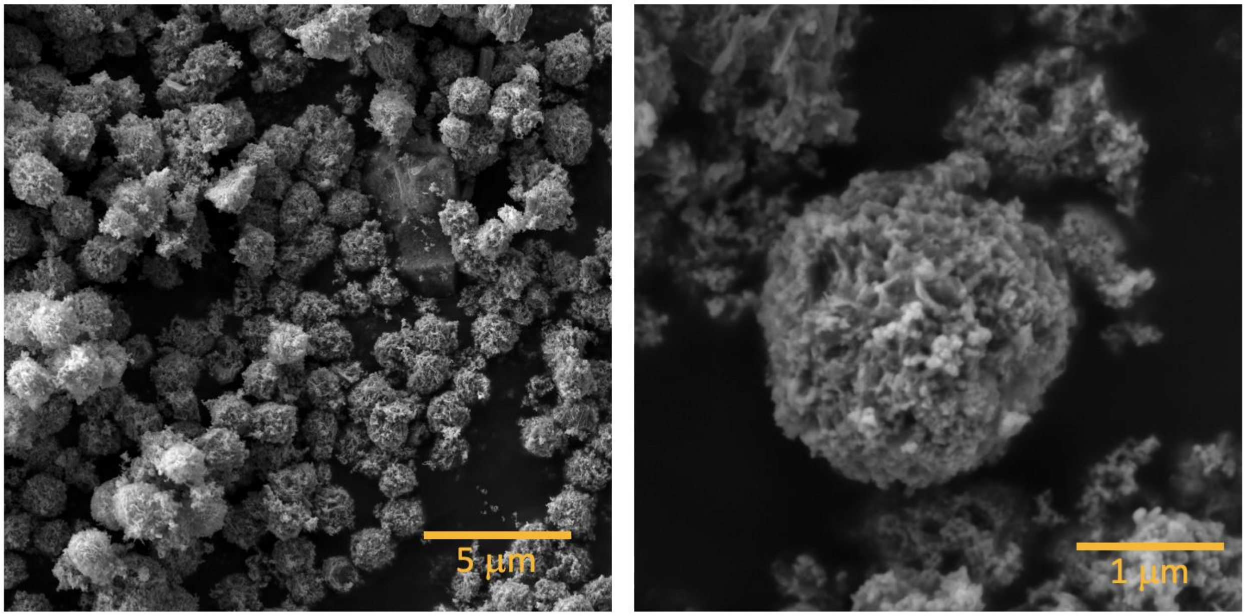

Figure S4: SEM images of Flower β-Bi_2O_3 after 4 photocatalytic cycles

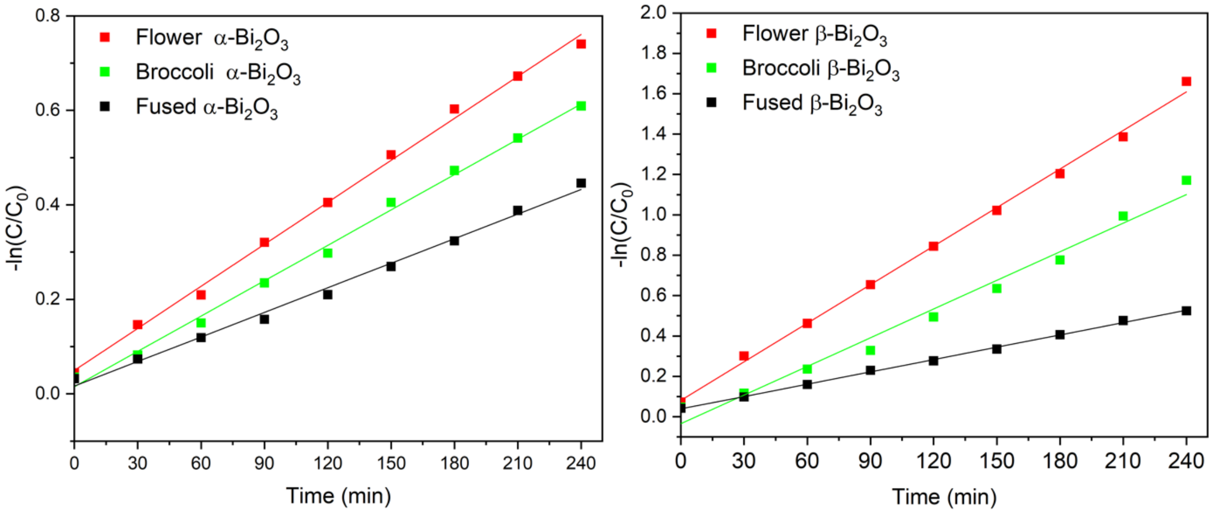

Figure S5: Kinetics of photocatalytic degradation of RhB using different catalysts

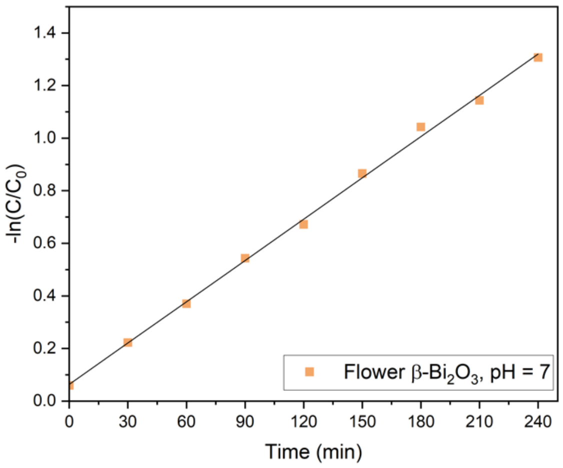

Figure S6: Kinetics of photocatalytic degradation of MO using flower β-Bi_2O_3
